# A 35-gene signature discriminates between rapidly- and slowly-progressing glioblastoma multiforme and predicts survival in known subtypes of the cancer

**DOI:** 10.1186/s12885-018-4103-5

**Published:** 2018-04-03

**Authors:** Azeez A. Fatai, Junaid Gamieldien

**Affiliations:** South African Bioinformatics Institute and SAMRC Unit for Bioinformatics Capacity Development, University of the Western Cape, Bellville, 7535, Western Cape, 7530 South Africa

**Keywords:** Glioblastoma multiforme, Prognostic genes, Risk groups, Chemoradiation resistance pathways

## Abstract

**Background:**

Gene expression can be employed for the discovery of prognostic gene or multigene signatures cancer. In this study, we assessed the prognostic value of a 35-gene expression signature selected by pathway and machine learning based methods in adjuvant therapy-linked glioblastoma multiforme (GBM) patients from the Cancer Genome Atlas.

**Methods:**

Genes with high expression variance was subjected to pathway enrichment analysis and those having roles in chemoradioresistance pathways were used in expression-based feature selection. A modified Support Vector Machine Recursive Feature Elimination algorithm was employed to select a subset of these genes that discriminated between rapidly-progressing and slowly-progressing patients.

**Results:**

Survival analysis on TCGA samples not used in feature selection and samples from four GBM subclasses, as well as from an entirely independent study, showed that the 35-gene signature discriminated between the survival groups in all cases (*p*<0.05) and could accurately predict survival irrespective of the subtype. In a multivariate analysis, the signature predicted progression-free and overall survival independently of other factors considered.

**Conclusion:**

We propose that the performance of the signature makes it an attractive candidate for further studies to assess its utility as a clinical prognostic and predictive biomarker in GBM patients. Additionally, the signature genes may also be useful therapeutic targets to improve both progression-free and overall survival in GBM patients.

**Electronic supplementary material:**

The online version of this article (10.1186/s12885-018-4103-5) contains supplementary material, which is available to authorized users.

## Background

Glioblastoma multiforme (GBM) is the most common and highly aggressive brain tumour. Patients with GBM have very poor prognosis, with the median OS time of 14.5 months [1]. Chemotherapy and radiotherapies are intended to improve patient survival, but are, however, hampered by development of resistance. Methylation of the promoter of the MGMT gene, which encodes O-6-methylguanine-DNA methyl-transferase, a DNA-repair enzyme that removes alkylating groups at the O6 of guanine residues, is a predictor of treatment response in GBM. Most studies that considered progression-free survival assessed only the prognostic value of MGMT promoter methylation [2–4]. Tumours with hypermethylated MGMT promoters are expected to benefit from temozolomide, an alkylating agent used for treating GBM, but reports regarding the prognostic value of this biomarker have been conflicting [5, 6].

Several gene expression prognostic and predictive signatures have been translated into clinical applications for cancer treatment. Oncotype DX is a 21-gene qRT-PCR assay used to predict likelihood of recurrence in women with estrogen receptor positive breast cancer [7, 8]. Mammostrat is prognostic immunohistochemical test that uses antibodies specific for SLC7A5, p53, HTF9C, NDRG1, and CEACAM5 to classify ER-positive, lymph node negative breast cancer cases into low-, moderate- or high-risk groups [9, 10]. Mammaprint is a 70-gene microarray-based test for predicting risk of metastasis in breast cancer [11].

In light of the lack of standardised prognostic biomarkers for GBM, we aimed to identify a mRNA expression derived prognostic signature using data from the Cancer Genome Atlas (TCGA - http://cancergenome.nih.gov/). As current prognostic feature selection approaches lack reproducibility and do not take chemoradioresistant pathways into consideration, we used a combination of pathway enrichment analysis and Support Vector Machine based Recursive Feature Elimination (SVM-RFE) to ensure that the genes selected as having predictive potential would also be biologically relevant to the phenoptype. We here describe a multigene signature that successfully predicts both progression-free and overall survival in glioblastoma multiforme.

## Methods

### Gene-centric expression data

Five hundred fifty eight GBM gene expression profiles generated by the Cancer Genome Atlas (TCGA) were downloaded from the NCI Genomic Data Commons Data Portal (https://portal.gdc.cancer.gov/projects/TCGA-GBM). Five hundred forty eight of the these profiles were obtained from GBM patients, and ten were from non-neoplastic patients. One profile was selected for each of the samples profiled two or more times. Five hundred twenty nine profiles left after removing those of non-neoplastic samples were used in this study (Additional file 1). The expression were profiled on Affymetrix HT HG-U133A platform. As gene expression of the TCGA samples was profiled in batches which could introduce bias in classification analysis [12], the statistical significance of batch effect was assessed as a function of the selected genes using guided Principal Component Analysis (gPCA) from the R package *gPCA* [13]. The approach used by TCGA (2008) [14] and Verhaak et al. (2011) [15] was employed to generate gene-centric expression data. The probe sequences of HT HG-U133A downloaded from Affymetrix were mapped against a database composed of RefSeq version 41 and GenBank 178 complete coding sequences using SpliceMiner [16]. Only perfect matches were considered and probes mapping to more than one gene were excluded. The output file from SpliceMiner and the HT HG-U133A chip definition file (cdf) were passed to the alternate cdf-generating function *makealtcdf* of AffyProbeMiner [17]. Probe sets with less than five probes were excluded from the resulting alternative cdf, which was then converted to an R package using *makecdfenv*. The cdf was used to perform Robust Multi-array Average normalization and summarization of the gene expression data, resulting in gene-centric data for 12161 genes.

An independent validation data set (GSE7696) profiled on HG-U133 Plus 2 Affymetrix platform and downloaded from the NCBI Gene Expression Omnibus (https://www.ncbi.nlm.nih.gov/geo/query/acc.cgi?acc=GSE7696) was equivalently treated. This data set contained gene expression data for 80 GBM and four non-neoplastic samples, and was chosen because of the availability of patients’ treatment information.

### Sample selection

**Fig. 1:**
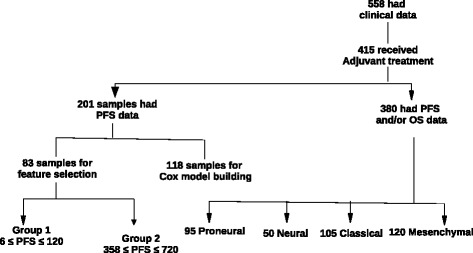
Sample selection for the identification of prognostic genes in glioblastoma multiforme. PFS: progression-free survival (days); OS: overall survival (days); adjuvant treatment: chemotherapy and radiation

To ensure that treatment did not introduce confounding effects, samples from patients that received adjuvant chemotherapy and radiation and had uncensored days to death or progression were selected. Figure 1 shows sample selection for the identification of genes with prognostic value. Four hundred fifteen patients received the standard GBM treatment. Semantically, tumour progression is a radiologically documented increase in tumour size after a subtotal surgical excision [18]. The time for this to occur is known as time to progression, which is the same as uncensored progression-free survival (PFS) [19]. Two hundred one patients had associated uncensored progression-free survival (PFS) times, and 380 had overall survival OS times (censored or uncensored).

Clinical data for all the patients used in this study were obtained from TCGA. PFS times for patients who experienced tumour progression within the follow-up period were obtained from the TCGA file for new tumour events. The GBM subtypes of samples used in our study were obtained from the supplementary clinical file provided by Brennan et al., (2013) [20].

There is no standard for classifying patients as rapid and slow GBM progressors after standard treatment. While the median PFS after treatment could be used as a separation point, it does not provide a ’buffer zone’ to filter out borderline samples close to the median that may fall in the incorrect group due to unknown confounding factors. Rather than defining an arbitrary exclusion range, we used the first (Q1) and third (Q3) quartiles, 120 and 341 days respectively, as boundaries to divide patients into three classes, since they are still dependent on the median and not influenced by extreme outliers. Class 1 contained 48 patients having PFS times between 6 and 120 days (rapidly-progressing) and class 2 contained 35 patients having PFS times between 358 and 720 days (slow progressing). Classes 1 and 2 were used in feature selection and the 118 remaining samples (Class 3) that fell within the inter-quartile range were used in PFS and OS analysis.

### Selection of genes discriminating between rapidly and slowly progressing GBM patients

In this present study, genes in the cancer-related pathways were considered in our feature selection because of their known roles in chemoradiation resistance, and to reduce the likelihood of selecting genes related to survival by chance. Studies have identified pathways and processes that drive resistance to chemotherapy and radiotherapy in cancer. Several of these genes are found in known cancer pathways [21–28]. Several genes in the NF- *κ*B and PI3K/Akt signaling pathways are associated with chemoresistance development in cancer [29, 30]. Also, genes involved in drug inactivation and efflux, DNA repair, and epithelial-mesenchymal transition have been shown to enhance drug resistance mechanisms [26, 31]. Pathway enrichment analysis was performed on the genes with high expression variance (median absolute deviation ≥ 0.5) across the 529 samples using the *Set Analyser* web service provided by the Comparative Toxicogenomics Database [32]. Genes were selected from the pathway categories related to cancer signaling pathways, reactive oxygen species metabolism, DNA repair, and drug transport and metabolism. A set of genes that discriminated between the rapidly-progressing and slowly-progressing groups were selected using a modified Support Vector Machine-Recursive Feature Elimination (SVM-RFE). SVM-RFE, proposed by [33], was modified by introducing 5-fold cross-validation into the SVM classifier step and capturing the error rate generated at this step (the figure showing the workflow for SVM-RFE is attached as Additional file 2).

### Survival analysis

The 118 Class 3 patients not used in the feature selection step were used to calculate regression coefficients (*β*) for the selected genes using univariate Cox proportional hazards analysis. The *β*’s were computed for the genes using *coxph* from the R survival package. Prognostic index, *PI*, was then calculated for each of the patients who received adjuvant chemotherapy and radiation and had PFS and/or OS data using the equation

$$PI = \beta_{1} *gene_{1} + \beta_{2} * {gene}_{2} + \ldots + \beta * {gene}_{g} $$

where *β*_*g*_ and *gene*_*g*_ are the regression coefficient and the gene expression value for gene g, respectively. Patients in Class 3 were classified into low-risk and high-risk groups by choosing a value between the highest and lowest PI that ensured proper patients distribution based on *PI*. Patients with *PI* scores greater or equal than the chosen value were assigned to the high-risk group, whereas those with *PI* scores less than the value were assigned to the low-risk group. 380 patients with OS times were also classified into low-risk and high-risk groups in the same way.

### Assessment of signature prognostic value in GBM subtypes

Verhaak et al. (2010) [15] identified four subtypes of GBM, namely proneural, neural, classical and mesenchymal, using gene expression data from 200 GBM samples. Brennan et al. (2013) [20] assigned additional 342 TGCA samples into the four subtypes using single-sample gene set enrichment analysis. A summarised clinical file provided by the authors was used in our study to assign patients to GBM subtypes. 95, 60, 105 and 120 of the 380 patients with available OS times were assigned to proneural, neural, classical and mesenchymal subtypes, respectively. 51, 33, 51 and 66 of the 201 patient group having associated PFS times were assigned to proneural, neural, classical and mesenchymal subtypes, respectively. We further categorised patients in each subtype into low-risk and high-risk groups.

### Assessment of signature prognostic value in an independent dataset

The prognostic value of the selected gene signature was validated with the data from patients in the Murat et al. [34] validation dataset who had primary tumours and received adjuvant chemo- and radiotherapy. *PI* was calculated for the patients using the *β*’s obtained from the TCGA training set and the expression values of the selected genes in the samples from the patients. They were classified into low-risk and high-risk groups in such a manner as to ensure proper patient distribution between the two groups. Survival of the low-risk and high-risk groups were determined for both the TCGA and validation cohorts using the Kaplan-Meier method. Differences in survival between the risk groups were estimated statistically by log rank test. Survival differences between groups was said to be statistically significant if *p*<0.05. Hazard ratios (HR) between risk groups were determined by Cox proportional hazards regression model.

### Mutivariate survival analysis to assess independent prognostic value

A multivariate Cox survival model was built using three variables: our prognostic index, MGMT promoter methylation, and age. Ages of patients at diagnosis were obtained from the clinical file provided by TCGA. MGMT promoter methylation status data were obtained from the clinical file provided by Brennan et al. [20] The univariate Cox analysis was first carried out on each variable followed by multivariate Cox analysis on all the variables. The coxph function in the R *survival* package was used for the analysis. Using the median *PI* value, the patients were assigned into low-risk or high-risk groups. Those with *PI* values lower than the median were assigned to low-risk groups, and those with *PI* to high-risk groups. The low-risk and the MGMT methylated promoter groups were used as references for prognostic index and MGMT promoter methylation status, respectively. Correlation of variables with PFS and OS was considered statistically significant at *p*(*Wald*)<0.05.

### Identifying functional interactions between signature genes

We used the STRING database of known and predicted protein-protein interactions (https://string-db.org/) [35] to construct an interaction network for the signature genes and to perform KEGG pathway enrichment analysis on the derived subnetwork.

## Results and discussion

In this present study, pathway-based and modified SVM-RFE-based methods were used to select a set of genes that discriminated between rapidly- and slowly-progressing GBM patients and combined into a signature. The prognostic value of the signature in predicting PFS and OS was accessed in the risk groups of GBM patients and validated on data set from an independent study. The independence of the signature in predicting PFS and OS was assessed by a multivariate Cox’s proportional hazards analysis. Studies on the identification of protein-coding multigene prognostic signatures in GBM focused on OS [7–9]. Overall survival (OS) is dependent on other factors besides gene expression. Progression-free survival, on the other hand, is expected to be a function of the expression of certain key genes. Genes whose expression across a cohort of patients correlated with OS were selected for survival analysis in these previous studies. This method has be shown to produce inconsistent signature genes in different data sets [36, 37].

### genes discriminate between rapidly- and slowly-progressing GBM patients

GBM is a highly aggressive brain tumour, and the median survival of patients with GBM is 14.6 months [38]. We hypothesized that the tumour’s pre-treatment expression of genes in pathways associated with chemoradioresistance in cancer would be predictive of how rapidly a GBM patient would experience progression after standard treatment. Signaling pathways (MAPK, JAK/STAT, WNT, NOTCH, Hedgehog, PIK3/AKT), cell cycle, drug transporters, reactive oxygen species metabolism and DNA repair system are known to be involved in chemoradioresistance in cancer [29, 39–41]. We also reasoned that PFS times were more appropriate than OS times in grouping patients. PFS times were expected to be more closely related to expression of key genes, while other factors including age and treatment after disease progression are also associated with OS.

**Fig. 2:**
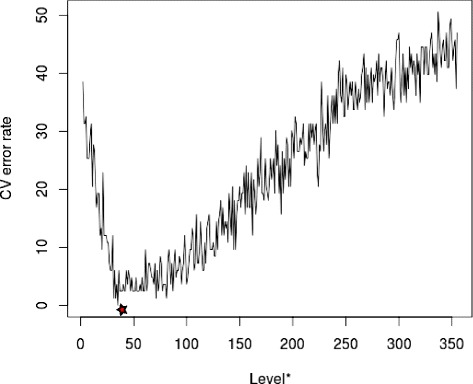
Cross-validated error rates of R-SVM in each recursive steps. *The number of features used for SVM classification in each step. Parameters for SVM: kernel = linear, cost = 10, and 5% cross-validation. The red star represents the level at which the minimal cross-validation error was achieved

Pathway enrichment analysis was performed on 3899 genes (Additional file 2) that had varied expression (*MAD*≥0.5) across 529 GBM samples. 18 of the 159 gene sets from the enrichment analysis were annotated for the known chemoradioresistance-associated pathways (Table 1). Assessment of batch effect in TGCA expression data set from 529 GBM samples as a function of the 356 genes extracted from the pathways (Additional file 3) showed that the data set did not have significant batch effect (*p*=0.118). Inspection of the unguided principal component analysis plot of the first two principal components also showed that no batch effect was present (Additional file 4). The extracted genes were used in gene selection by the modified SVM-RFE. Our modified SVM-RFE was used to identify genes that discriminated between 48 rapidly-progressing patients (between 6 and 120 days PFS) and 35 slowly-progressing patients (between 358 and 720 days PFS). Figure 2 shows the plot of 5-fold cross-validation error rate against number of genes at each recursive step, starting with the 356 genes extracted from the pathways. The CV error rate decreased with decreasing number of genes until it reached 35 genes, which discriminated between rapidly- and slowly-progressing GBM patients at 100% accuracy. Further decreases in the number of genes resulted in increasing error rate.

**Table 1:** Selected pathway categories associated with chemoradiation resistance by pathway enrichment analysis on genes with high expression variance

Pathway	Number of genes
Cell cycle	62
MAPK signaling	87
p53 signaling	39
WNT signaling	48
Glutathione metabolism	23
TGF- *β* signaling	30
Insulin signaling	40
ErbB signaling	29
Phosphatidylinositol signaling	25
Mismatch repair	12
Inositol phosphate metabolism	20
JAK-STAT signaling	22
Apoptosis	25
VEGF signaling	22
Nucleotide excision repair	15

The PFS times and expression levels of selected genes in the 118 Class 3 patients were used in multivariate Cox regression analysis to compute *β*’s for the genes. Table 2 shows the *β*’s calculated for the 35 selected genes. *PI* scores were calculated for all patients who received adjuvant chemotherapy and radiation (380) by substituting *β*’s and expression levels of selected genes into the prognostic index formula. The scores were then used to classify samples into low- and high-risk groups in survival analysis.

**Table 2:** Correlation of the expression of the 35 signature genes with progression-free survival using univariate Cox model

Gene	Contained pathway	Coefficient (*β*)	HR	p
*ABL1*	Cell cycle	-0.1215	0.886	0.650
	ErbB pathway			
*CCNA1*	Cell cycle	-0.0899	0.914	0.520
*CCND1*	Cell cycle	0.0746	1.077	0.620
*CCNE1*	Cell cycle	-0.4975	0.608	0.032
*CDC6*	Cell cycle	0.2324	1.262	0.410
*CDK2*	Cell cycle	-0.5268	0.591	0.150
*CDKN1C*	Cell cycle	-0.0285	0.972	0.900
*CDKN2A*	Cell cycle	0.1415	1.152	0.170
	p53 pathway			
*DKK1*	WNT pathway	0.1759	1.192	0.040
*FZD3*	WNT pathway	0.2434	1.276	0.230
*FZD7*	WNT pathway	0.4934	1.638	0.022
*GADD45G*	MAPK pathway	0.2850	1.330	0.200
*GHR*	JAK-STAT pathway	-0.2292	0.795	0.180
*GSTT1*	Glutathione metabolism	0.0485	1.050	0.590
*HSPA1B*	JAK-STAT pathway	-0.1244	0.883	0.340
*ID4*	TGF- *β* pathway	-0.1680	0.845	0.350
*IGFBP3*	p53 pathway	0.1103	1.117	0.340
*INHBB*	TGF- *β* pathway	-0.1187	0.888	0.390
*IRS2*	Insulin signaling	-0.0665	0.936	0.790
*LIFR*	ErbB pathway	-0.1009	0.904	0.670
*PDGFRA*	MAPK pathway	0.0262	1.027	0.760
*PIK3CA*	Phosphatidylinositol signaling	0.1479	1.159	0.420
*PLA2G5*	Phosphatidylinositol signaling	-0.0176	0.983	0.900
*POLE3*	Nucleotide excision repair	0.0615	1.063	0.840
*PPARGC1A*	MAPK pathway	0.4121	1.510	0.045
*PRKAR2B*	Insulin signaling	0.0641	1.066	0.660
*PYGB*	Insulin signaling	-0.4071	0.666	0.160
*SFRP1*	WNT pathway	-0.1216	0.886	0.270
*SFRP4*	WNT pathway	0.1461	1.157	0.270
*SH2B2*	Insulin signaling	-0.0199	0.980	0.950
*STAG3L4*	JAK-STAT pathway	-0.0001	1.000	1.000
*STMN1*	MAPK pathway	0.0837	1.087	0.750
*THBS2*	Focal adhesion	-0.1319	0.876	0.330
*THBS3*	Focal adhesion	-0.3916	0.767	0.160
*VEGFA*	VEGFA signaling	-0.0268	0.974	0.810

All the seed pathways in Table 1 except mismatch repair had at least one representative in the signature. Cell cycle had the highest number of genes (eight), followed by WNT pathway, which had five. The expression of four of the selected genes were significantly correlated with PFS (*p*<0.05): *DKK1*, *FZD7*, and *PPARGC1A* showed positive correlation (*β*>0), and *CCNE1* displayed negative correlation (*β*<0) (Table 2).

### Several signature genes are linked to survival in other cancers

Several genes in the signature have been reported to be associated with progression-free and/or overall survival in other cancers. DKK1, FZD3, FZD7, SFRP1, and SFRP4 are regulators of the Wnt/ *β* pathway. Overexpression of DKK1 is predictive of unfavourable overall survival and time to recurrence in intrahepatic cholangiocarcinoma patients [42]. Overexpression of FZD3 in colorectal patients was correlated with poor survival [43]. Underexpression of SFRP1 is associated with poor survival and may be an independent predictive and prognostic factor for prostate cancer [44]. SFRP4 increased the sensitivity of ovarian cancer cell lines to cisplatin, suggesting it is a predictive marker of chemoresistance in the cancer [45]. CCNA1, CCND1, CCNE1, CDC6, CDK2, CDKN1C and CDKN2A regulate the cell cycle. CCND1 amplication was associated with poor prognosis in estrogen receptor positive breast cancer [46] and [47] found it to be an independent prognostic factor in primary tumours and metastases as well as an independent prognostic factor in metastasis. CDC6 expression was correlated with overall and recurrence survival in non-small cell lung cancer patients [48]. CDKN2A promoter methylation was correlated with poor prognosis of colorectal cancer patients [49, 50]. CDK2, regulated by CDKN2A, is a known oncogene and regulator of the cell cycle. Its regression coefficient (*β*<0) in our study, however, showed that it was positively associated with progression-free survival. Its overexpression was associated with shorter survival in oral cancer [51]. GADD45G is implicated in stress signaling responses to physiological or environmental stressors, resulting in cell cycle arrest, DNA repair, cell survival and senescence, or apoptosis [52, 53]. GADD45G methylation and protein expression were independently associated with survival of gastric cardia adenocarcinoma patients [54] and esophageal squamous cell carcinoma patients [55].

### The 35-gene signature predicts progression-free and overall survival in both TCGA and independent dataset

**Fig. 3:**
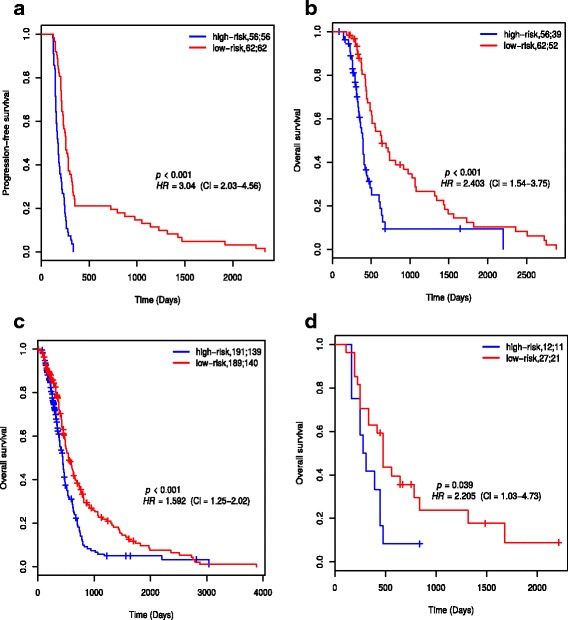
Kaplan-Meier plots for low-risk and high-risk groups of GBM patients that received adjuvant chemotherapy and radiotherapy. The patients were classified based on PI score. **a** PFS plots and **b** OS plots of risks groups from 118 TCGA patients not used in the feature selection. **c** OS plots of risk groups from 380 TCGA patients with OS times. **d** OS plots of risks groups from the Murat et al. data set used for validation. The two numbers in the topright corner of each plot represents the total number of patients in each risk group and the number of patients who experienced progression or death within the follow-up periods, respectively

The 35 genes that discriminated between rapidly- and slowly-progressing patients were combined into a signature and its prognostic value first assessed in the patients that were not used in the feature selection step (Class 3). The prognostic index (PI) scores of these patients were standardized and used to split the patients into low- and high-risk groups. Figures 3a and 3b show the PFS and OS Kaplan Meier plots, respectively, for the two prognostic groups. The median PFS and OS times for the low-risk group (256 days, 95% CI = 232 - 299 days and 635 days, 95% CI = 502 - 1024 days) were significantly higher than those of the high-risk group (175 days, 95% CI = 158 - 204 days and 393 days, 95% CI = 345 - 454 days) (*p*<0.05).

Two hundred seventy nine of the 380 patients who received adjuvant chemotherapy and radiotherapy died before the end of the follow-up period. The remaining 101 patients were alive at the end of follow-up or were lost to follow-up. The 380 patients were split into low- and high-risk groups. Figure 3c shows the OS plots for these prognostic groups. There was a statistically significant difference in OS between the groups (*p*<0.05). The median OS time (548 days, 95% CI = 486 - 646) of the low-risk group was significantly higher than that (442 days, 95% CI = 394 - 476) of the high-risk group (*p*<0.05).

Thirty nine patients in the validation cohort received adjuvant chemotherapy and radiation. The *β*’s computed with the TCGA cohort and the expression levels of the signature genes in the validation cohort were used to calculate *PI* scores for the patients in the validation cohort. The patients were then split into low- and high-risk groups. The median OS of the low-risk group was higher than that of the high-risk group, and the difference in OS between the groups was statistically significant (*p*<0.05) (Fig. 3d).

The results show that the 35-gene signature identified from the TCGA dataset may be a generically applicable predictor of progression-free and overall survival in GBM, since prognostic value in the prediction of overall survival was validated in an independent cohort.

### The 35-gene signature predicts progression-free and overall survival in four GBM subtypes

**Fig. 4:**
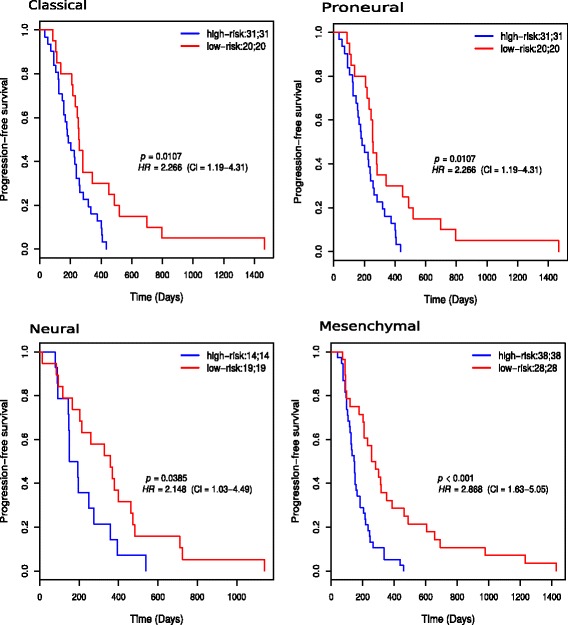
Kaplan-Meier progression-free survival plots for risk groups of patients in each subtype of GBM. The patients were classified based on PI score. The two numbers in the topright corner of each plot represents the total number of patients in each risk group and the number of patients who experienced progression or death within the follow-up periods, respectively

The prognostic value of the signature in predicting PFS and OS in subtypes of GBM was assessed. 51, 51, 3 and 66 patients belonged to the classical, proneural, neural, and mesenchymal subtypes, respectively. Figure 4 shows the results of the PFS survival analysis in the subtypes. There was statistically significant difference in survival between low- and high-risk groups in all the subtypes (*p*<0.05). In the classical subtype, the median PFS times of low- and high-risk groups were 256 and 186 days respectively. In the mesenchymal subtype, the median PFS times were 269 and 146 days respectively. In the neural subtype, the median PFS times were 358 and 172 days, respectively. In the proneural subtype, the median PFS times were 304 and 172 days, respectively.

**Fig. 5:**
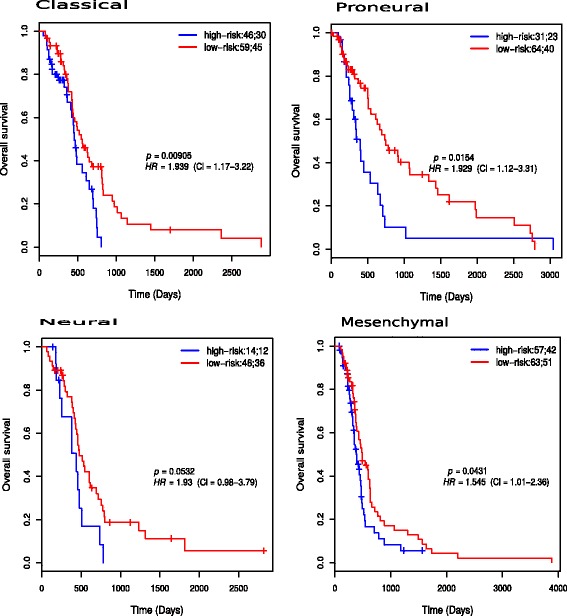
Kaplan-Meier overall survival plots for risk groups of patients in each subtype of GBM. The patients were classified based on PI score. The two numbers in the topright corner of each plot represents the total number of patients in each risk group and the number of patients who experienced progression or death within the follow-up periods, respectively

One hundred five classical, 95 proneural, 60 neural and 120 mesenchymal subtype patients were used for subtype-specific OS analysis. Figure 5 shows the Kaplan-Meier OS plots for high-risk and low-risk groups in each subtype. The low- and high-risk groups differed significantly in OS in all the subtypes (*p*<0.05). In the classical subtype, the median OS times of low- and high-risk groups were 544 and 452 days respectively. In the mesenchymal subtype, the median OS times were 485 and 394 days respectively. In the neural subtype, the median OS times were 476 and 435 days, respectively. In the proneural subtype, the median OS times were 748 and 395 days, respectively. Reports from previous studies show that the prognostic value of MGMT promoter methylation in GBM patients is controversial. Zhang et al. [56] showed that MGMT promoter methylation was associated with better PFS and OS in patients with GBM regardless of therapeutic intervention, and associated with longer OS in GBM patients treated with alkylating agents. Costa et al. [5] did not find significant association between MGMT promoter methylation and the outcome of Portuguese GBM patients treated with temozolomide. Brennan et al. [20] however reported that MGMT promoter methylation was only correlated with OS in the GBM classical subtypes. The possible explanation for these conflicting reports on the prognostic value of MGMT promoter methylation could thus be due to differences in the GBM subtype distribution which was not considered in most previous studies. Our 35-gene signature, however, predicted PFS and OS regardless of the subtype, suggesting that it may be a more effective predictor of overall and progression-free survival in GBM.

### The 35-gene signature is an independent predictor of PFS and OS in GBM patients

A multivariate Cox regression model analysis involving the prognostic index, age and MGMT promoter methylation was carried to assess the independence of the gene signature to predict PFS and OS. 79 TCGA GBM patients had associated days to progression, and age and MGMT promoter methylation status (38 methylated and 41 unmethylated) data. Two hundred sixty nine patients had days to death and age and MGMT promoter methylation (135 methylated and 134 unmethylated) data. The results from the univariate and multivariate analyses on the three variable are shown in Table 3. MGMT promoter methylation was not correlated with PFS in both univariate and multivariate Cox analyses (*p*>0.05). Prognostic index, age and MGMT promoter methylation were significantly correlated with OS in the univariate and multivariate analyses (*p*<0.05). The univariate Cox’s proportional hazard analysis showed that age and the prognostic index based on the 35-gene signature were both significantly correlated with PFS (*p*<0.05), but only the prognostic index was significantly correlated with PFS in the multivariate analysis (*p*<0.05). This showed that the expression signature is an independent predictor of PFS and OS in GBM patients.

**Table 3:** Univariate and multivariate Cox’s proportional hazards model analyses of prognostic factors for progression-free and overall survival

Variable	Progression-free survival				Overall survival							
			Univariate		Multivariate				Univariate		Multivariate	
	n	n ^∗^	HR	*p(Wald)*	HR	*p(Wald)*	n	n ^∗^	HR	*p(Wald)*	HR	*p(Wald)*
Prognostic groups^1^	79	79	3.41	3.00E-6	3.13	2.97E-5	269	181	1.63	1.45E-3	1.60	2.46E-3
High-risk^2^												
Age	79	79	1.02	4.30E-2	1.01	3.70E-1	269	181	1.04	3.50E-8	1.03	6.12E-7
MGMT methylation status	79	79	1.33	2.14E-1	1,26	3.20E-1	269	181	1.68	6.59E-4	1.52	7.35E-3
Unmethylated^3^												

^∗^Number of events; ^1^high-risk and low-risk groups; ^2^low-risk was used as reference; ^3^methylated was used as reference

Post-treatment tumour progression depends largely on alterations in classical cancer and chemotherapy/radiation resistance-related pathways. This is supported by findings from the multivariate Cox’s proportional hazard analysis findings as only the 35-gene prognostic index was significantly associated with PFS and was an independent predictor of PFS. Overall survival, on the other hand, is determined by many factors. Age at diagnosis is one of the most important factors associated with overall survival in cancer and has been demonstrated in GBM [57–59]. While the prognostic value of MGMT promoter methylation in GBM remains controversial, our findings showed that prognostic index, age and MGMT promoter methylation are all independent prognostic factors for overall survival.

### Signature genes belong to a functional interaction subnetwork enriched for known cancer pathways

**Fig. 6:**
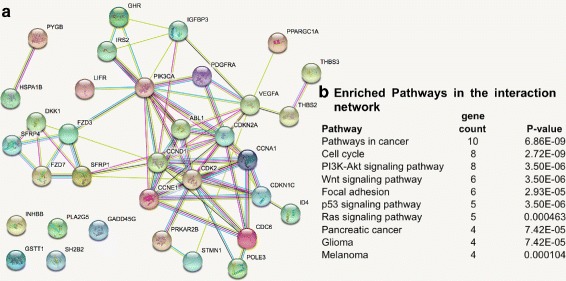
Analysis of the subnetwork formed from the interaction between signature genes. **a** The subnetwork from the STRING database. **b** Enriched pathways in the subnetwork

A subnetwork generated from the interactions between the signature genes had significantly more interactions than would be expected for a random set of proteins of similar size (PPI enrichment *p*=1.11×10^−16^) (Fig. 6). The network was also significantly enriched (*p*<0.01) for KEGG cancer pathways and pathways known to drive tumour initiation and progression, such as the cell cycle and PI3K-Akt, Wnt, p53 and Ras signaling [60, 61].

### A subset of the signature genes may be relevant to GBM biology and may have utility in drug discovery

Combinatorial medicine have been proposed for the treatment of tumour recurrence. It involves therapeutically targeting as many genomic alterations responsible for a disease in a patient as possible and has strong implications for overcoming the challenge of tumour progression and drug resistance [62, 63]. One of the ways to overcome this challenge is to prioritise combinations of genes to be targeted based on their unique roles in tumour progression. Of the signature genes, only *ABL1, CCND1, CCNE1, PDGFRA, PIK3CA* were found to be linked to predisposition to at least one cancer by the Online Mendelian Inheritance in Man (OMIM) database [64]. However, *CCNA1, CDK2, CDKN1C, CDKN2A, FZD3, HSPA1B, IGFBP3, PDGFRA, PIK3CA, PLA2G5, THBS2* and *VEGFA* all have gene ontology annotations related to apoptosis, while *ABL1, FZD7, PDGFRA, PIK3CA, SFRP1, THBS2*, and *VEGFA* are annotated as being involved in angiogenesis (data not shown). Collectively this may indicate differential gene expression explicitly directed towards towards resisting induced cell death by both intrinsic and extrinsic factors and optimising the tumour microenvironment for maximum fitness. This, combined with the knowledge that the signature genes are involved in classical pathways implicated in cancer drug resistance, suggests that the highlighted genes should be further validated and assessed as drug targets in designing novel combinatorial therapies for GBM in future studies.

## Conclusion

We propose that the performance of the signature makes it an attractive candidate for further studies to assess its utility as a clinical prognostic and predictive biomarker in GBM patients, and that its component genes may also have utility as therapeutic targets for improving both progression-free and overall survival.

## Additional files

Additional file 1 Workflow of the modified SVM-RFE used for selecting a set of genes that discriminated between rapidly-progressing and slow-progressing GBM patients. (XLSX 66.7 kb)

Additional file 2 Genes with high expression variance used in pathway enrichment analysis. (PDF 23.5 kb)

Additional file 3 Unguided principal component analysis to identify batch effect in the TCGA data set as a function of genes from chemoradioresistance-associated pathways. *p*=0.118, indicating absence of significant batch effect in the data. Samples in each batch are denoted by a different colour and symbol. (TXT 2.05 kb)

Additional file 4 Unguided principal component analysis to assess batch effect. (PDF 161 kb)
