# Supplementary figures and images for: A 35-gene signature discriminates between rapidly- and slowly-progressing glioblastoma multiforme and predicts survival in known subtypes of the cancer
Source: BMC Cancer. 2018 Apr 3;18:377. doi: 10.1186/s12885-018-4103-5 (PMC5883543; doi:10.1186/s12885-018-4103-5)

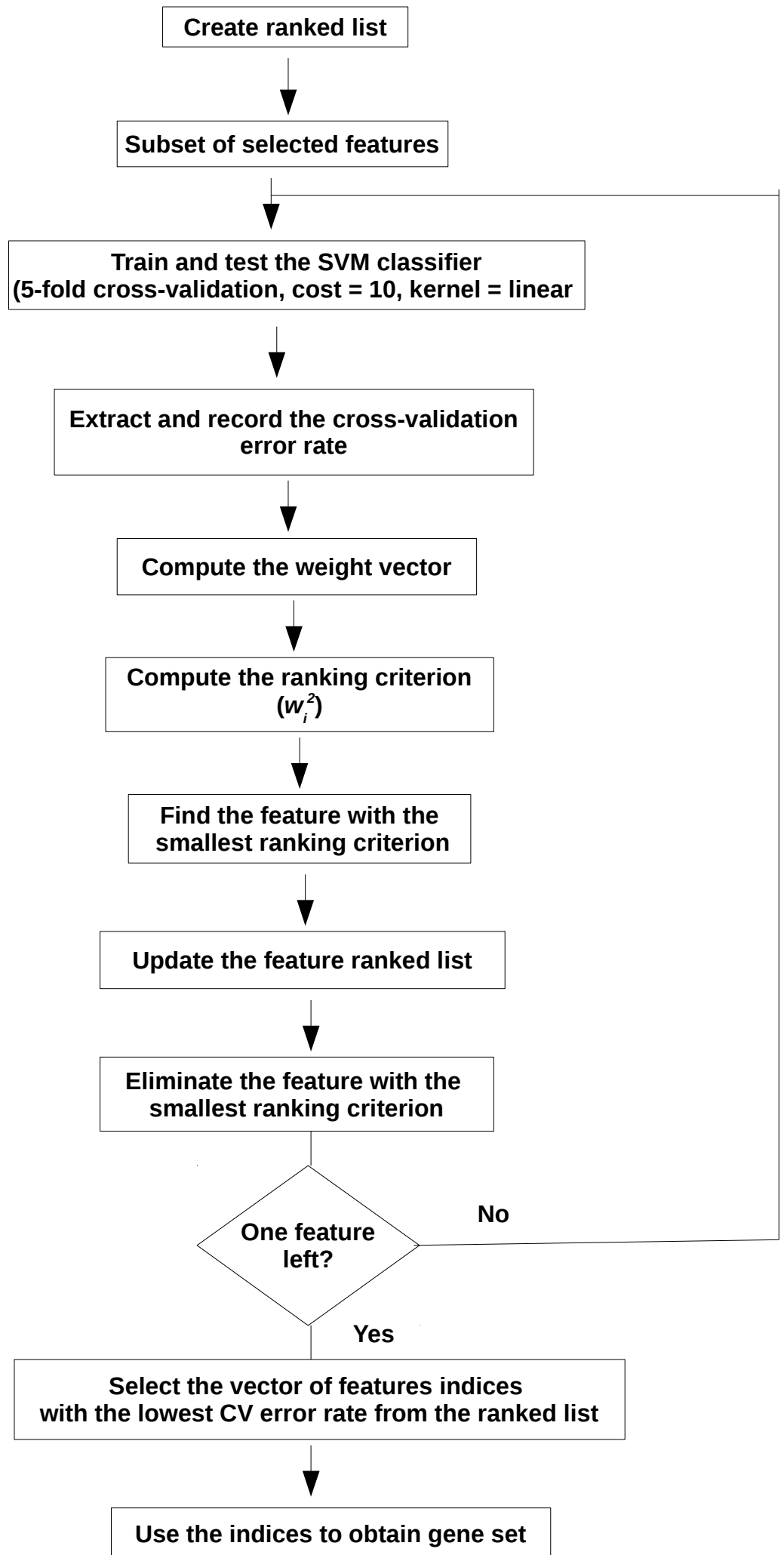

Supplement: Supplementary file 2 — Genes with high expression variance used in pathway enrichment analysis. (PDF 23.5 kb) [file 12885_2018_4103_MOESM2_ESM.pdf]

**Distribution of delta values from permutations**  
**(delta=7.408; p-value=0.118; filt=5000)**

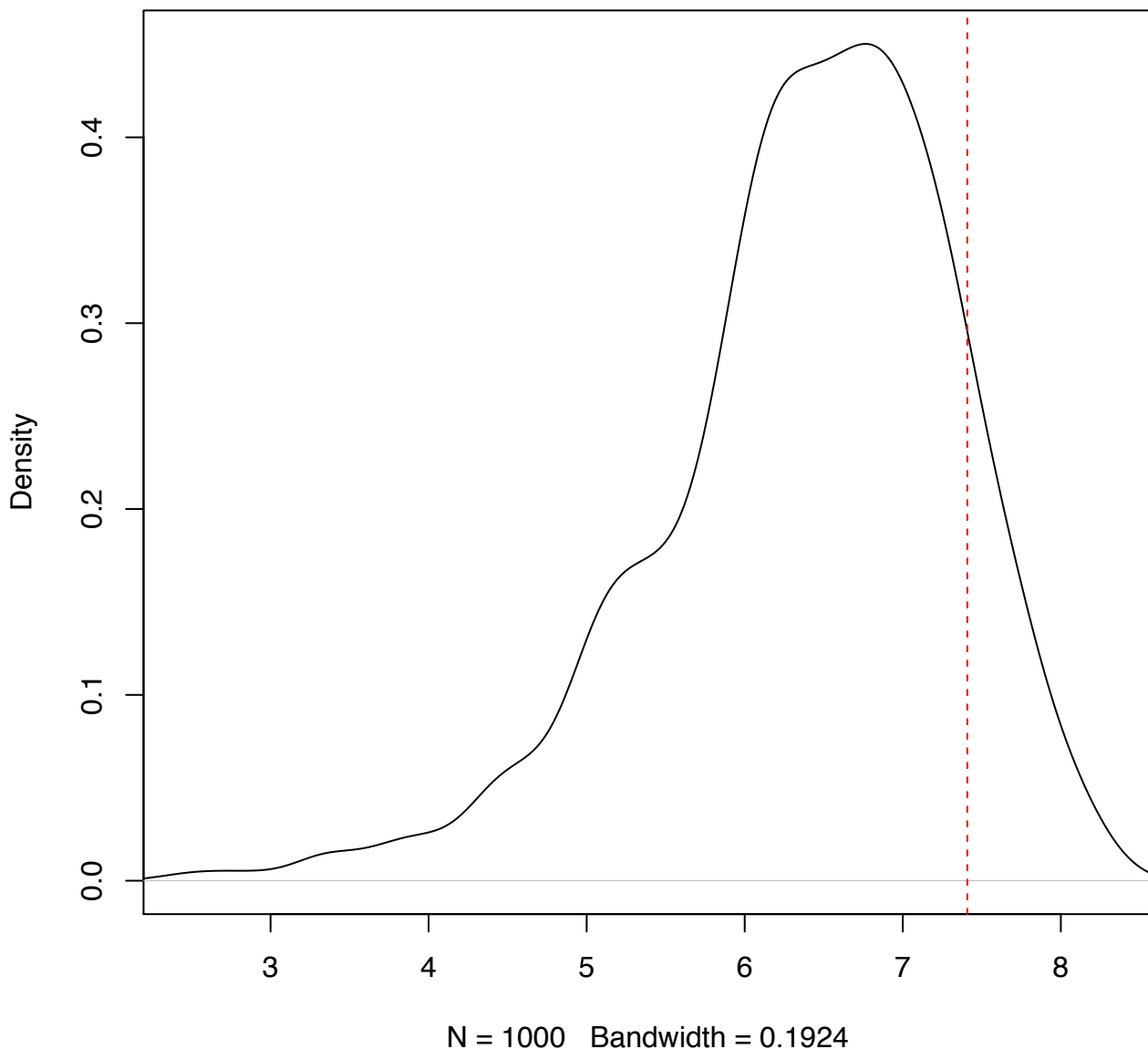

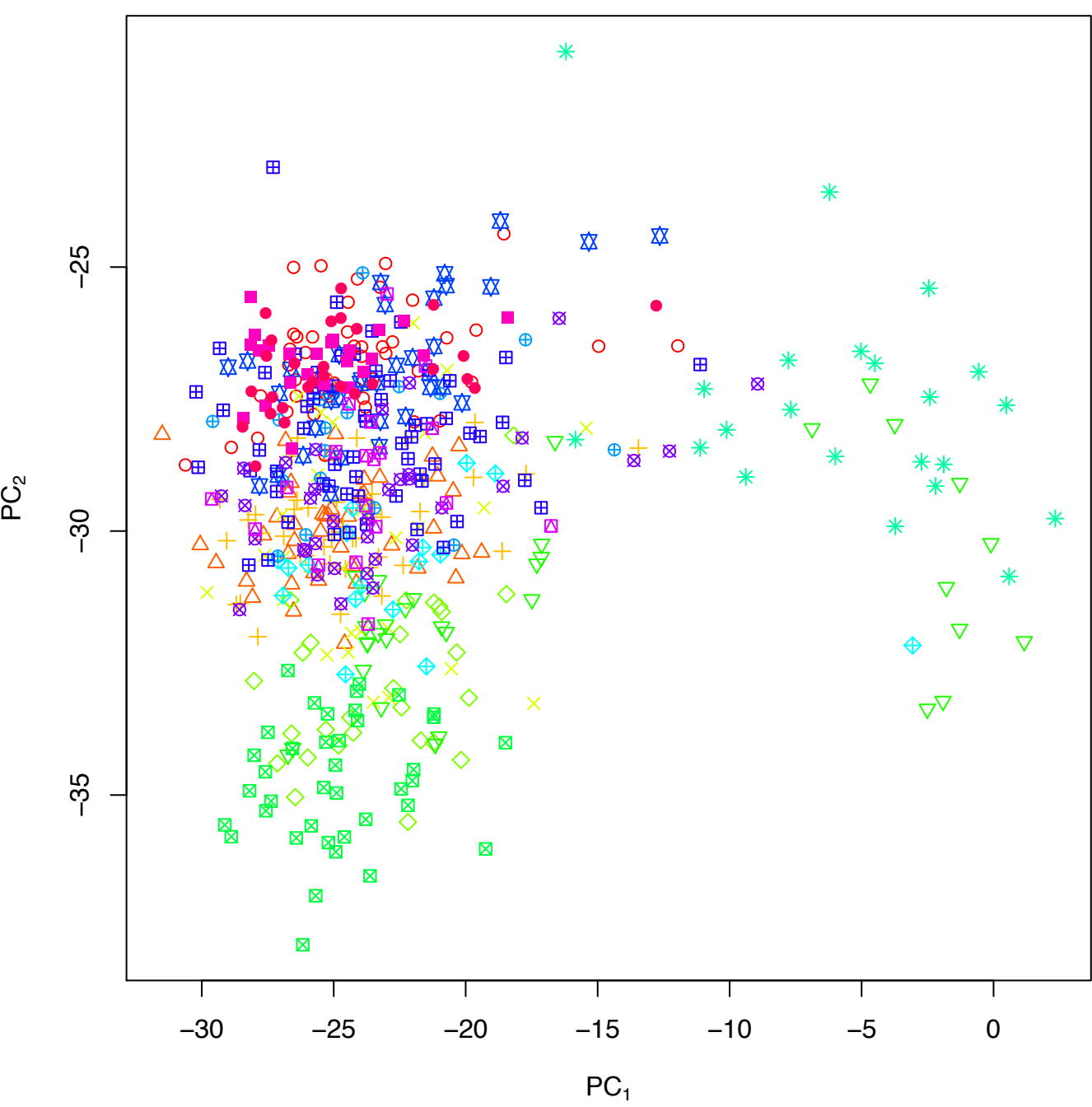

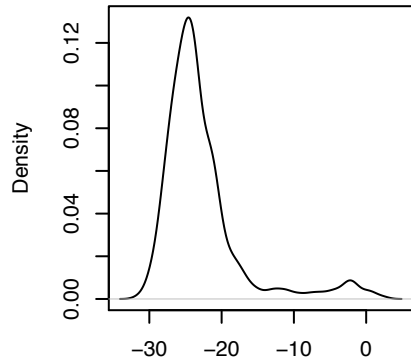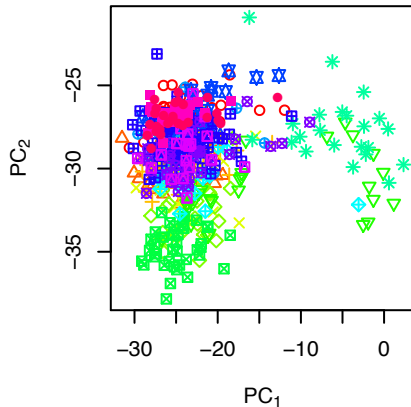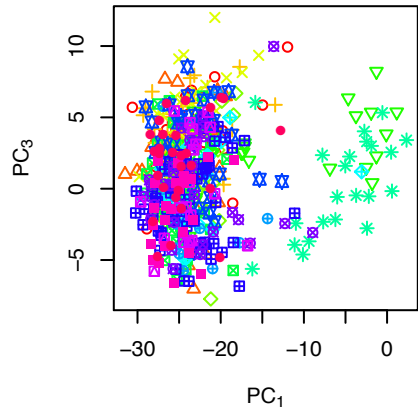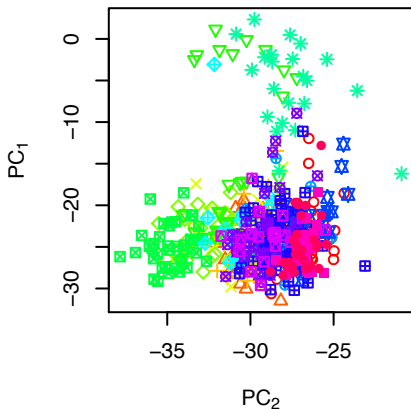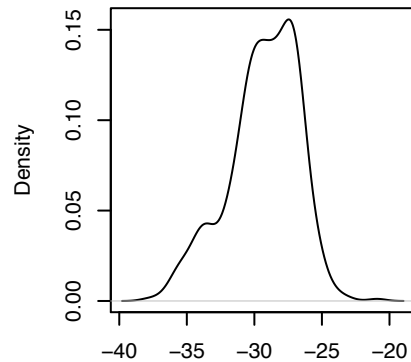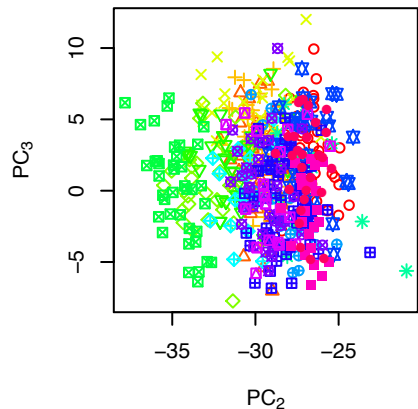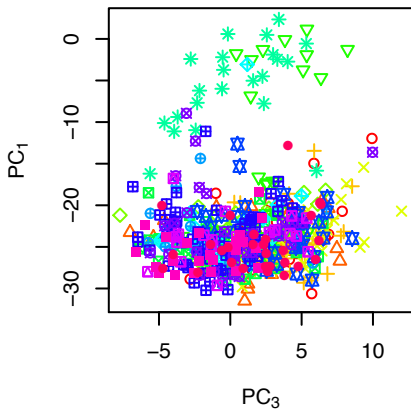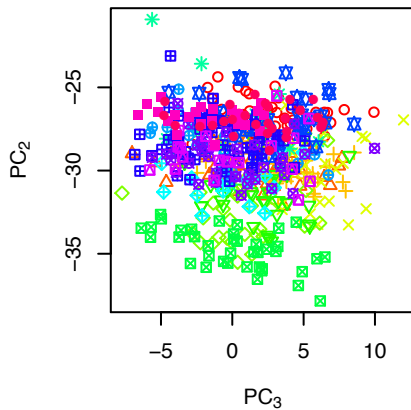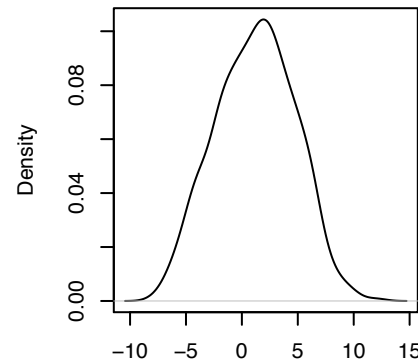

Supplement: Supplementary file 4 — Unguided principal component analysis to assess batch effect. (PDF 161 kb) [file 12885_2018_4103_MOESM4_ESM.pdf]
